# Cross-Cultural Validation and Psychometric Evaluation of the Birth Beliefs Scale Among Portuguese Pregnant Women

**DOI:** 10.1177/01939459261466136

**Published:** 2026-08-04

**Authors:** Marlene Lopes, Teresa Neves, Margarida Vieira, Alexandrina Cardoso

**Affiliations:** 1 Health Sciences Research Unit: Nursing (UICISA: E), Nursing School of Coimbra, University of Coimbra, Portugal; 2 Centre for Interdisciplinary Research in Health, Faculty of Health Sciences and Nursing, Universidade Católica Portuguesa, Porto, Portugal; 3 Nursing School, RISE-Health, University of Porto, Portugal

**Keywords:** childbirth beliefs, pregnant women, psychometric validation, cross-cultural adaptation, birth beliefs

## Abstract

**Background:**

Beliefs about childbirth shape women’s expectations, decisions, and experiences. Although several tools assess related constructs such as fear or self-efficacy, few instruments focus specifically on childbirth beliefs. The Birth Beliefs Scale (BBS) addresses this gap by measuring 2 dimensions: beliefs in childbirth as a natural event and as a medical event.

**Objective:**

We sought to adapt the BBS to the Portuguese context and evaluate its psychometric properties.

**Methods:**

This methodological study involved cross-cultural adaptation and psychometric testing. The adaptation followed internationally recognized guidelines, including translation, synthesis, back-translation, expert review, and pretesting. Psychometric evaluation included exploratory and confirmatory factor analysis. The sample comprised 241 pregnant women enrolled in childbirth preparation programs in central Portugal.

**Findings:**

The original two-factor structure was partially confirmed following the removal of 3 items with low psychometric performance, including 2 related to labor pain. The Portuguese version (BBS-pt) demonstrated acceptable internal consistency for the Natural subscale (α = 0.68) and lower consistency for the Medical subscale (α = 0.58), suggesting cultural and contextual nuances in how birth beliefs are expressed.

**Conclusion:**

The BBS-pt is a promising instrument for assessing birth beliefs among Portuguese women. While further refinement is needed, particularly regarding pain-related items, the scale provides a valuable foundation for both research and clinical practice. It may assist nurse-midwives and other maternity care providers in tailoring antenatal education and intrapartum support to women’s individual belief patterns, thereby contributing to more personalized and woman-centered care.

## Introduction

Childbirth is widely recognized as one of the most profound and transformative events in a woman’s life. It shapes identity, self-confidence, and maternal competence, influencing the transition to motherhood in both positive and negative ways.^1-3^ Expectations formed during pregnancy strongly predict women’s satisfaction with birth and are closely linked to maternal and neonatal outcomes.^4,5^

Birth beliefs encompass cultural, emotional, spiritual, and psychological perceptions and expectations related to pregnancy, labor, and childbirth. They shape how women and families understand, prepare for, and experience birth.^6^ In some narratives and professional paradigms, childbirth is framed as a dangerous event requiring medical control, whereas in others it is regarded as a physiological, social, and spiritual rite of passage grounded in trust in the body and environment.^5,7^ These beliefs are central to women’s experiences of childbirth and guide their attitudes, decisions, and behaviors during labor and delivery.

Women’s childbirth decisions are shaped by cultural, historical, and social contexts, as well as by interactions with healthcare professionals, significant others, and media portrayals. Contemporary narratives often emphasize risk and scrutinize non-conventional choices, which can undermine women’s autonomy. This dynamic contributes to high rates of medical intervention, even in low-risk pregnancies, as observed in many Western countries.^7,8^ Childbirth is commonly conceptualized along a spectrum, from a physiological to a medical process, or as a blend of both.^9-11^ Women who view childbirth as natural and physiological tend to trust their bodies, perceive pain as manageable, and prefer fewer medical interventions due to concerns about cascading effects. In contrast, women who view childbirth as medical often perceive labor as risky, emphasize technology and safety, and more readily accept interventions such as pharmacological analgesia and cesarean delivery.^8,9,12-14^ Although the natural and medical perspectives are often framed as opposites, most women hold nuanced beliefs along a continuum. These beliefs influence their choice of care providers, birth settings, delivery methods, and pain management strategies.^9,15,16^ Women leaning toward a physiological view often seek skills that enhance self-efficacy, whereas those aligned with a medical view are more likely to delegate control to professionals. For the former, concerns center on autonomy and authority, while for the latter, risk and pain predominate in decision-making.^9^ Beliefs about childbirth are dynamic, shaped by psychological factors such as optimism, anxiety, and self-perceived competence, and may shift with personal experiences.^17^ Birth experiences often reinforce or challenge pre-existing beliefs: medicalized births may weaken physiological beliefs, whereas medical views are often strengthened.^18^ Importantly, alignment between expectations and experiences tends to reinforce prior beliefs, underscoring the need for individualized care that respects these perspectives.

The medicalization of low-risk pregnancies increases healthcare costs and may expose women to unnecessary interventions. It can also contribute to disrespect and mistreatment during childbirth.^7^ Conversely, women who experience fewer interventions report more positive long-term outcomes.^19,20^ In recognition of this, the World Health Organization (WHO) highlights the promotion of positive childbirth experiences as a universal priority.^21^

Existing tools measure constructs such as childbirth self-efficacy, locus of control, and decision-making, but few focus specifically on childbirth beliefs. The Birth Beliefs Scale (BBS), developed and validated in Israel by Preis and Benyamini,^11^ addresses this gap. It captures 2 dimensions: childbirth as a natural event and childbirth as a medical event. Understanding these beliefs is essential to design personalized, evidence-based interventions that align with women’s perspectives and support positive experiences.

## Purpose

This study aimed to translate, culturally adapt, and evaluate the psychometric properties of the European Portuguese version of the BBS. Contextualizing the instrument for Portuguese pregnant women is expected to expand understanding of childbirth beliefs and the sociocultural factors shaping them. The validation of the Portuguese version of the BBS may strengthen nurse-midwifery knowledge and support the development of person-centered, evidence-based interventions that prepare women for childbirth and promote positive maternal outcomes.

## Methods

This methodological study was conducted in 2 phases: (1) cross-cultural adaptation of the original BBS and (2) evaluation of the psychometric properties of the Portuguese version.

### Cross-Cultural Adaptation

The adaptation process followed the five-step methodology,^22^ ensuring semantic, cultural, and conceptual equivalence. A literature review and team discussions first examined the theoretical relevance of the construct for the Portuguese context. Birth beliefs were defined as cultural, emotional, spiritual, and psychological perceptions and expectations regarding pregnancy and childbirth, shaping how women and families prepare for and experience birth. Authorization to translate and adapt the BBS into European Portuguese was obtained from the original authors.

*Step 1: Translation*. Two independent native Portuguese translators, fluent in English, produced separate translations. Their aim was semantic equivalence while preserving meaning.
*Step 2: Synthesis*. The research team reviewed and merged both versions, resolving minor grammatical discrepancies.
*Step 3: Back-translation*. Two independent native English speakers, blinded to the original, back-translated the Portuguese version. This confirmed fidelity to the original content.
*Step 4: Expert panel*. An expert panel (2 PhD nursing researchers and 1 nurse-midwife with >20 years of clinical experience) evaluated cultural relevance, semantic equivalence, idiomatic appropriateness, and readability. Based on consensus, Version 1 of the Portuguese BBS was finalized.
*Step 5: Pretest*. Eighteen pregnant women attending Childbirth Preparation Programs completed Version 1. They provided feedback on clarity, comprehensibility, and semantic accuracy through questionnaires and follow-up interviews, which supported semantic and experiential equivalence.

### Psychometric Properties

#### Participants

A convenience sample of 241 pregnant women was recruited from 9 Community Care Units in central Portugal between September 2023 and September 2024. Sample size was based on the BBS’s 11 items and a 10:1 participant-to-item ratio, requiring at least 110 participants.^23^ Inclusion criteria were pregnant women aged ≥18 years, in the third trimester of a low-risk pregnancy, enrolled in childbirth preparation programs at participating community care units, and able to read and speak Portuguese. Nurse-midwives at community care units invited eligible women during childbirth preparation program sessions. Participation was voluntary, and declining had no impact on care. Consenting participants received questionnaires with written instructions and could respond either on paper (sealed envelopes) or online via QR code. Ethical approval was obtained from the Ethics Committee of the Central Regional Health Administration in June 2023 (no. 52/2023).

#### Instruments

The questionnaire comprised 2 sections: (1) sociodemographic and obstetric data (age, education, employment, household composition, gestational age, and parity); and (2) the Portuguese version of the BBS, consisting of 11 items rated on a 5-point Likert scale (1 = strongly disagree to 5 = strongly agree). The original BBS includes 2 subscales: BBS-Natural (items 3, 5, 7, 8, and 11) and BBS-Medical (items 1, 2, 4, 6, 9, and 10). Scores are calculated as means, with higher values indicating stronger beliefs in the respective dimension. In the original validation study,^11^ the internal consistency of these subscales was assessed at 2 points during pregnancy, with Cronbach’s alpha coefficients ranging from 0.70 to 0.82.

### Data Analysis

Psychometric evaluation included Exploratory Factor Analysis (EFA) and Confirmatory Factor Analysis (CFA). EFA was conducted using the principal components method with Varimax rotation, based on the correlation matrix. Factor retention was guided by eigenvalues >1 and the scree plot. In addition, given the bifactorial structure of the original scale, a 2-factor solution was imposed to test the stability of the factorial model. For CFA, univariate normality was assessed through skewness (Sk) and kurtosis (Ku), with |Sk| < 3 and |Ku| < 10 considered acceptable for maximum likelihood estimation. Outliers were identified using Mahalanobis squared distance (*D*^2^). Minimal missing values were replaced by the mean of the corresponding variable. Model fit was evaluated using χ^2^/df < 5, CFI and GFI >0.90, and RMSEA <0.08. Model selection was informed by the lowest MECVI value, and additional adjustments were made based on modification indices (MI > 11, *P* < .001) when theoretically justified. Reliability was examined through composite reliability (CR) and Cronbach’s alpha (α), with values ≥0.70 considered satisfactory. Construct validity was assessed across 3 domains: (a) convergent validity, with AVE >0.50; (b) discriminant validity, confirmed when AVE exceeded the squared inter-factor correlation; and (c) factorial validity, with standardized loadings (λ > 0.50) and item reliability (λ^2^ > 0.25). Slightly lower values were accepted, as is common in social sciences.

Analyses followed validation guidelines by DeVellis and Thorpe.^24^ Descriptive statistics and EFA were performed in SPSS v29.0 (IBM Corp., Armonk, NY, USA), and CFA with invariance testing in AMOS v22.0 (IBM Corp., Armonk, NY, USA).

## Results

The results address 2 key aspects: the cross-cultural adaptation of the instrument and the assessment of its psychometric properties.

### Cross-Cultural Adaptation

The translation and adaptation process followed a rigorous, standardized methodology comprising forward translation by 2 independent bilingual translators, synthesis of the translations, back-translation by 2 other translators, expert committee review, and pretesting with pregnant women. This procedure ensured semantic, idiomatic, experiential, and conceptual equivalence between the original and Portuguese versions.

Although the clarity of the original instrument facilitated translation and most items were easily adapted, 2 concepts required more in-depth analysis and discussion. First, the English term *birth* may refer both to giving birth (woman-centered) and to the child’s moment of birth (child-centered). To reflect the intended woman-centered meaning of the scale, *birth* was translated as the Portuguese word *parto*, which specifically denotes “the act or process of giving birth” and thus better captures women’s beliefs about the experience rather than the moment of expulsion alone. Second, the literal translation of *medical event (evento médico)* was considered too vague. It was therefore replaced with *evento que requer cuidados e supervisão médica* (“event requiring medical care and supervision”), which more accurately conveys the intended construct. Pretest interviews with 18 pregnant women confirmed the clarity, accuracy, and cultural relevance of the Portuguese version, with no reported difficulties in comprehension. In summary, the cross-cultural adaptation produced a Portuguese version of the BBS that is linguistically accurate, culturally appropriate, and conceptually equivalent to the original.

### Psychometric Properties

#### Sample Characteristics

The study included 241 pregnant women attending Childbirth Preparation Programs at 9 Community Care Units in central Portugal between September 2023 and September 2024. Participants ranged in age from 20 to 45 years, with a mean of 32.2 years (SD = 4.7). Gestational age varied from 21 to 41 weeks, with a mean of 33.5 weeks (SD = 3.8). Most participants were nulliparous (72.6%), and 21.2% had experienced one previous birth. Educational attainment was generally high: 65.1% held higher education degrees, of whom 41.9% had a bachelor’s degree and 22.0% a master’s degree. Only 4.1% reported less than secondary education. Nearly all participants (91.3%) were employed, primarily in salaried positions (79.7%). Almost the entire sample (96.3%) cohabited with the baby’s father. No variables had missing values, resulting in a complete dataset for analysis (Table 1).

**Table 1.** Participant Characteristics (N = 241).

Characteristic	n (%)	Mean ± SD	Range
Age (years)		32.2 (4.8)	20-45
Gestational age (weeks)		33.5 (3.8)	21-41
Educational qualifications			
Less than secondary	10 (4.1)		
Secondary	74 (30.7)		
Bachelor’s	101 (41.9)		
Master’s	53 (22.0)		
Doctorate	3 (1.2)		
Employment Status			
Self-employed	28 (11.6)		
Salaried	192 (79.7)		
Unemployed	19 (7.9)		
Student	2 (0.8)		
Household Composition			
Lives alone	5 (2.1)		
With partner	163 (67.6)		
With partner + child(ren)	65 (27.0)		
With other relatives	4 (1.7)		
With partner + relatives	4 (1.7)		
Number of Previous Births			
None	175 (72.6)		
One	51 (21.2)		
Two	12 (5.0)		
≥Three	3 (1.2)		

#### Exploratory Factor Analysis

The total sample of 241 women was randomly subdivided, resulting in a subsample of 97 women for the EFA, while the CFA was conducted with a subsample of 144 women.

Based on the 97 participants included in the EFA, the descriptive analysis of item scores indicated adequate psychometric sensitivity for factor extraction. The sample adequacy for factor analysis was assessed using the Kaiser-Meyer-Olkin (KMO) measure, which, although moderate (KMO = 0.64), was considered acceptable. Bartlett’s test of sphericity was statistically significant (*P* < .001), supporting the relevance of performing an EFA.

Although the initial unforced solution revealed a four-factor structure explaining 64.38% of the total variance, the emergent factors lacked theoretical coherence and did not align with the conceptual framework of the original instrument. To preserve consistency with the original scale, a two-factor solution was therefore imposed, which accounted for 43.27% of the total variance. In this forced structure, some items presented low communalities, specifically items 2, 4, 6, and 8, with values below 0.30, indicating limited contribution to the extracted factors. Nonetheless, the rotated factor pattern was theoretically interpretable and supported the plausibility of the original bifactorial model. Overall, the items showed factor loadings above 0.35 on a single factor, suggesting a suitable factorial structure, largely consistent with the original model, except for item 6. This item exhibited a factor loading below 0.30 and similar cross-loadings on both factors (Table 2). Such dispersion of factor loadings may indicate that the item lacks sufficient discriminatory power and does not clearly align with either of the latent dimensions identified in this solution. Therefore, the removal of item 6 is suggested, and a CFA is deemed essential to test the adequacy of this factorial structure.

**Table 2.** Factor Loading From the Exploratory Factorial Analysis (n = 97).

Item	Mean ± SD	Component 1	Component 2
1. Birth is a medical event *O parto é um evento que requer cuidados e supervisão médica*	4.53 ± 0.70		0.74
2. Nowadays, there is no reason why women should suffer pain in childbirth *Hoje em dia, não há razão para as mulheres sofrerem com dor durante o trabalho de parto*	3.86 ± 1.04		0.36
3. A woman’s body knows how to give birth *O corpo de uma mulher sabe como fazer nascer o filho*	3.98 ± 0.79	0.75	
4. There are many things that can go wrong during childbirth *Há muitas coisas que podem correr mal durante o parto*	3.98 ± 0.76		0.50
5. Labor should be allowed to proceed at its own pace *O trabalho de parto deve evoluir ao seu próprio ritmo*	4.18 ± 0.66	0.77	
6. Often, a woman’s body structure does not allow her to give birth naturally *Frequentemente, a estrutura corporal da mulher não lhe permite parir de forma natural*	2.60 ± 1.08	−0.26	0.21
7. Birth is a natural event *O parto é um evento natural*	4.07 ± 0.71	0.84	
8. Pain in childbirth is a significant part of the birth experience *A dor é uma parte significativa da experiência de parto*	3.65 ± 0.87	0.41	
9. Childbirth requires vigilant medical supervision *O parto requer vigilância clínica contínua*	4.21 ± 0.78		0.73
10. Childbirth is a dangerous process *O parto é um processo perigoso*	3.12 ± 0.98		0.62
11. Birth is an empowering experience *O parto é uma experiência empoderadora*	4.05 ± 0.74	0.62	
Variance (%)		24.93%	18.34%

#### Confirmatory Factor Analysis

**Figure 1.**
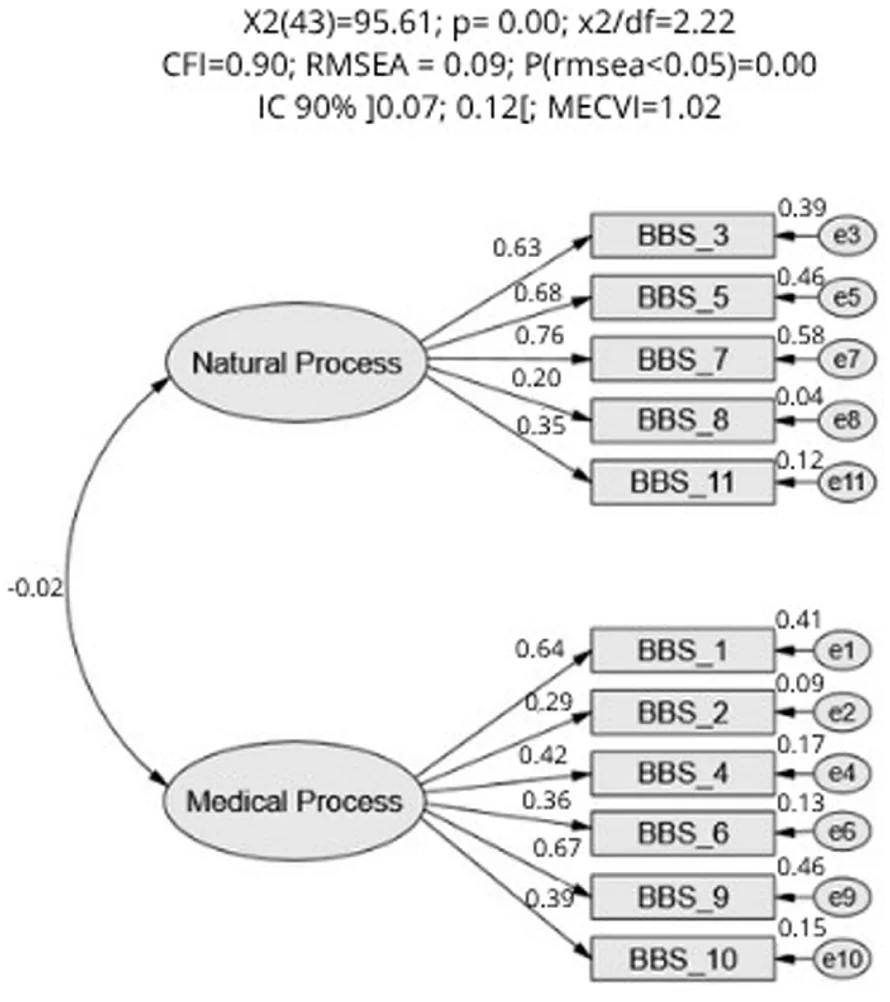
Factor structure of the Portuguese version of the BBS, based on the original model. BBS, Birth Beliefs Scale.

**Figure 2.**
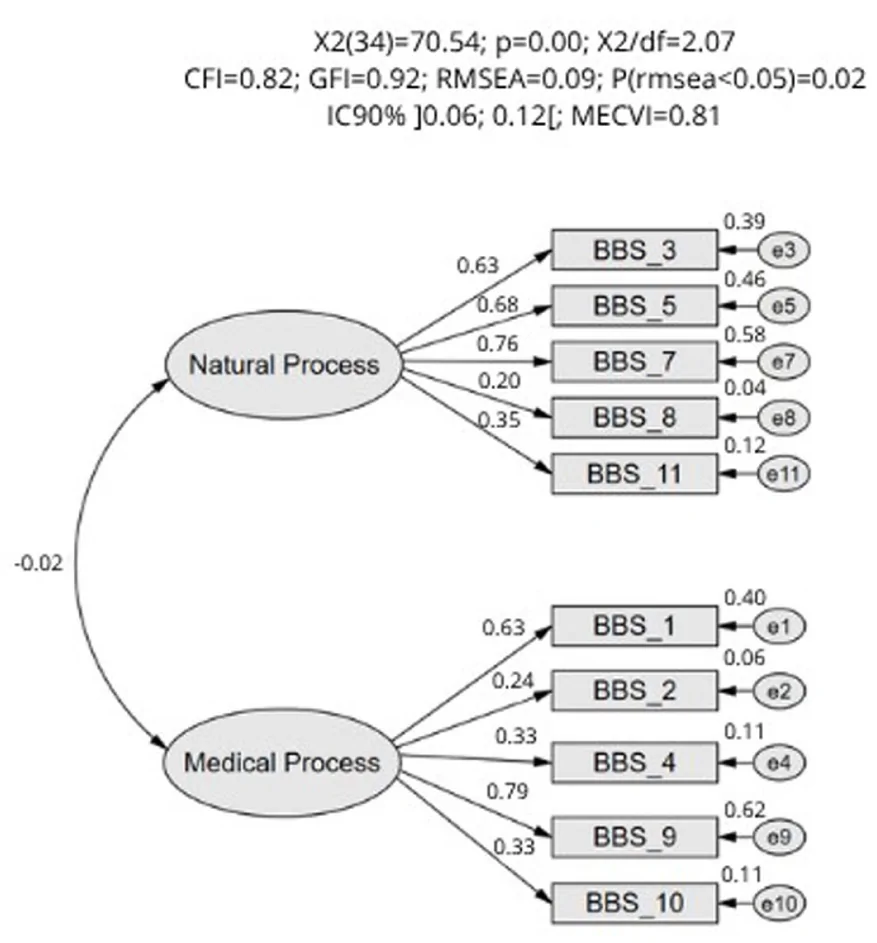
Factor structure of the Portuguese version of the BBS, based on the EFA-derived model. BBS, Birth Beliefs Scale; EFA, Exploratory Factor Analysis.

The CFA was conducted with a sample of 144 women to assess model fit, comparing the original bifactorial model (Figure 1) with the bifactorial model derived from the EFA (Figure 2). Based on the goodness-of-fit indices, the EFA-derived model showed a better fit to the data than the original model, and was therefore retained for further analysis.

The analysis revealed an overall acceptable model fit across most indices (χ^2^/df = 2.07; CFI = 0.82; GFI = 0.92; RMSEA = 0.09; MECVI = 0.81). Standardized factor loadings (λ) and individual item reliability (λ^2^) were generally adequate, although some items showed lower-than-expected loadings. Item 2 (“Nowadays, there is no reason for women to suffer pain during labor”) and item 8 (“Pain is a meaningful part of the childbirth experience”) presented very low factor loadings (λ < 0.30) and were therefore removed from the model. Items 4, 10, and 11 also had loadings below the previously established threshold, but were retained in the model as part of a conservative strategy.

**Figure 3.**
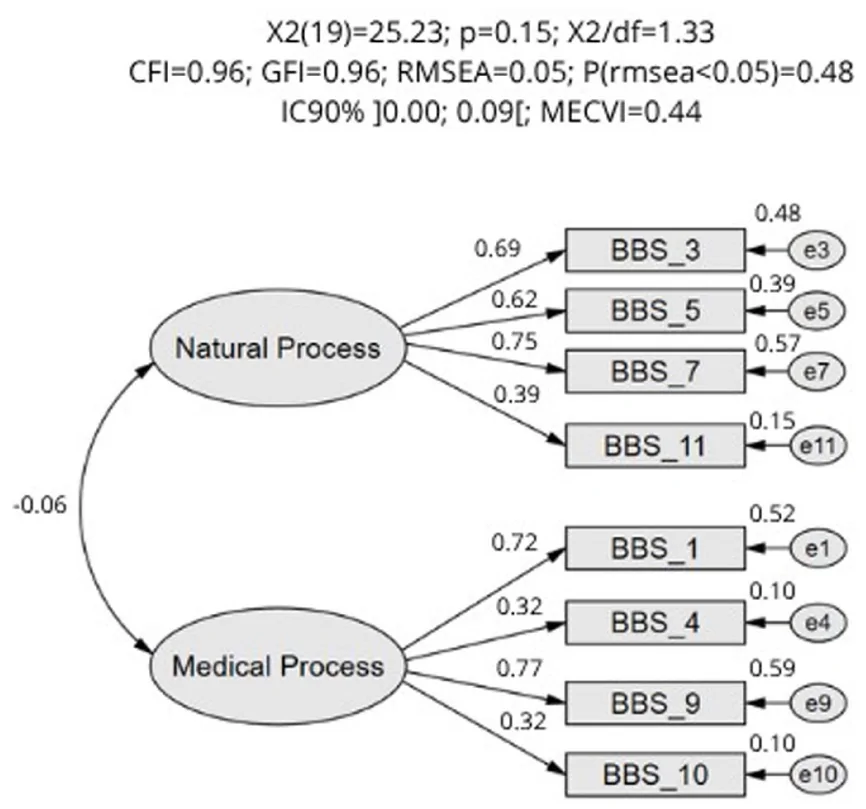
Refined factor structure of the Portuguese version of the BBS. BBS, Birth Beliefs Scale.

All items met the criteria for normality. However, multivariate outliers were identified (*P*1 and *P*2 < .001), leading to a revised analysis after excluding 2 observations with high Mahalanobis distance (*D*^2^). The reanalysis showed improved model fit (χ^2^/df = 1.33; CFI = 0.96; GFI = 0.96; RMSEA = 0.05; MECVI = 0.44), as presented in Figure 3.

The final model demonstrated a significantly better fit to the data compared to the initial structure (χ^2^_[15]_ = 45.31, *P* < .05), with a lower MECVI value (MECVI: 0.81 vs 0.44).

Construct reliability, as shown in Table 3, was adequate for the Natural Process dimension, but lower for the medical process dimension (CR and Cronbach’s α < 0.70). Standardized factor loadings ranged from 0.32 to 0.77, and individual item reliability ranged from 0.10 to 0.59. Regarding convergent validity, AVE values were below the desired threshold (AVE ≥ 0.50). However, comparison of AVE with the squared correlations between factors supported discriminant validity.

**Table 3.** Construct Reliability, Convergent Validity, and Discriminant Validity of the BBS Factors (Refined Model).

Factors	Number of items	CR	α	AVE	ρ^2^
Natural Process	4	0.71	0.68	0.39	0.004
Medical Process	4	0.63	0.58	0.33	0.004

Abbreviations: α, Cronbach’s alpha; ρ^2^, squared correlation between factors; AVE, average variance extracted; BBS, Birth Beliefs Scale; CR, composite reliability.

## Discussion

The BBS was cross-culturally adapted to the Portuguese context following the guidelines of Beaton et al.^22^ Initial EFA revealed unstable four-component structures lacking theoretical coherence. A forced two-factor solution, consistent with the original conceptual model distinguishing Natural and Medical beliefs, produced a more interpretable and theoretically grounded structure.

Item 6 *(“Often, a woman’s body structure does not allow her to give birth naturally”)* did not load significantly on either factor and was excluded from the final version. This item may not have been well understood by Portuguese participants, either due to ambiguous phrasing or underlying assumptions about anatomical adequacy and childbirth capacity, which may not resonate strongly within Portuguese cultural or maternity care narratives. It is also possible that the notion reflects a medicalized perspective that women enrolled in childbirth preparation programs were less familiar with or resistant to, given the emphasis on physiological birth in these settings.

Items 2 *(“Nowadays, there is no reason for women to suffer from pain during labor”)* and 8 *(“Pain is a significant part of the childbirth experience”)* also demonstrated low loadings in both EFA and CFA and were removed. Although they represent opposing perspectives on labor pain, one aligned with biomedical discourse advocating for pharmacological relief and the other with a physiological framing of pain as intrinsic to childbirth, both items showed limited variability and mid-range mean scores (3.86 and 3.65). Responses clustered around agreement (51% for item 8; 62.6% for item 2), reducing their discriminatory power. This pattern points to cultural ambivalence or dual conceptualization of labor pain among Portuguese women. On the one hand, item 2 reflects a modern medicalized view consistent with widespread availability of epidural analgesia in Portugal. On the other, item 8 resonates with a physiological perspective that interprets pain as meaningful and integral to childbirth, a view promoted by woman-centered models of care. The coexistence of these perspectives suggests that women may simultaneously normalize and medicalize labor pain, acknowledging it as natural while also expecting relief. This duality blurs the boundary between Natural and Medical beliefs and weakens the coherence of pain-related items in factor analysis.

Evidence from other contexts supports this interpretation. In a US mixed-method study with a diverse sample, 95% of respondents believed women have the right to pain relief during childbirth, yet 68% acknowledged the value of labor pain and 87% agreed it should be clinically managed.^25^ Such findings illustrate the complex and sometimes contradictory nature of beliefs about labor pain. In Portugal, where epidural use is common, pain may be perceived as both expected and avoidable. The predominance of nulliparous women in this sample (72.6%) may have further contributed to neutral or inconsistent responses, as their beliefs are likely shaped more by social narratives, health professionals, and antenatal education than by lived experience, and they may not yet hold firmly defined views about labor pain. Taken together, these findings suggest that pain-related beliefs may be particularly sensitive to cultural and experiential influences. The exclusion of pain items raises important questions about whether labor pain can be conceptualized as a stable belief dimension across cultures or whether it should be treated as a distinct, context-dependent construct.

Another possible explanation for the poor performance of the pain-related items lies in how they were interpreted. Despite careful translation and pretesting, some items may still have lacked semantic clarity or cultural resonance. For example, item 2 *(“Nowadays, there is no reason for women to suffer from pain during labor”)* may have been interpreted less as reflections of personal belief and more as general or policy-oriented statements, expressing technocratic or compassionate ideals. As a result, women may have responded in a neutral or disengaged way, rather than expressing their own views. Given the subjective and culturally specific nature of pain-related beliefs, future adaptations of the BBS should consider reformulating such items to prompt explicitly personal perspectives rather than socially normative positions. Doing so may enhance psychometric performance and better capture the multidimensional nature of childbirth pain beliefs. These results indicate that labor pain may not fit neatly within a binary framework of “natural” versus “medical” beliefs. Rather, it may constitute an independent dimension reflecting ambivalence between its physiological meaning and the expectation of relief. Developing a separate pain subdomain could strengthen the theoretical and psychometric coherence of the instrument.

Women’s beliefs about labor pain are shaped by a complex interplay of physiological, psychological, social, cultural, and spiritual factors. Literature increasingly shows that labor pain is a unique and meaningful experience influenced by cognitive, social, and environmental conditions. When interpreted as purposeful and supported within a safe environment, pain can become empowering. Conversely, when perceived as meaningless or threatening, it often generates distress and reliance on medical relief. Interventions that frame pain as intentional and manageable, while strengthening coping resources and providing continuous support, may transform it into a more positive component of childbirth.^26,27^ These insights challenge a strictly dualistic conceptualization of pain beliefs. Women may hold beliefs along a continuum that integrates both physiological and biomedical understandings, shaped by values, experiences, and cultural norms.

To date, only 2 studies have examined the BBS outside Israel. The scale was originally developed and validated in Israel by Preis and Benyamini,^11^ who reported a clear two-factor structure distinguishing beliefs about childbirth as a Natural versus a Medical event, with acceptable to good internal consistency for both subscales. Cronbach’s α ranged from 0.70 to 0.76 for the Natural beliefs subscale and from 0.79 to 0.82 for the Medical beliefs subscale across 2 measurement points during pregnancy. The Turkish validation, conducted with pregnant women, confirmed the two-factor structure and demonstrated excellent psychometric properties, including high internal consistency (α = 0.89 for Natural beliefs; α = 0.87 for Medical beliefs) and strong test–retest reliability (ICC > 0.98).^28^ In comparison, the reliability coefficients observed in the Portuguese sample were lower, particularly for the Medical subscale (α = 0.68 for Natural beliefs; α = 0.58 for Medical beliefs).

A Dutch study, in contrast, applied the BBS to maternity care professionals and focused on content and discriminant validity.^29^ While not directly comparable to studies with women, it provides useful insights by showing how beliefs vary across professional roles and clinical contexts. Obstetricians scored higher on Medical beliefs, while community midwives scored higher on Natural beliefs. Such findings highlight the BBS’s potential to reveal how cultural, professional, and institutional factors shape childbirth beliefs.

The present study expands this body of work by validating the BBS among Portuguese pregnant women. The original two-factor structure was partially supported, though 3 items with poor performance were removed. Internal consistency was acceptable for the Natural subscale (α = 0.68) but lower for the Medical subscale (α = 0.58), likely reflecting both the reduced number of items and cultural heterogeneity in how childbirth is conceptualized. These results reinforce the importance of cross-cultural validation, suggesting that while the underlying model remains relevant, contextual adaptation is needed to improve clarity and robustness. Comparison with other applications of the BBS reveals methodological and cultural variation in its performance. Table 4 summarizes findings across Israel, Turkey, the Netherlands, and Portugal.

**Table 4.** Cross-Cultural Applications of the Birth Beliefs Scale: Populations, Psychometric Methods, and Main Findings.

Study (year)	Country/Language	Population	Sample size	Factor structure	Internal consistency (α)	Test–retest reliability
Preis and Benyamini (2017)^11^	Israel/Hebrew	Pregnant women	850	2-factor structure (Natural, Medical)	Natural: 0.70-0.76 Medical: 0.79-0.82	Natural: 0.89 Medical: 0.94
Paker and Ertem (2020)^28^	Turkey/Turkish	Pregnant women	205	2-factor structure confirmed by EFA and CFA	Natural: 0.89 Medical: 0.87	Natural: 0.99 Medical: 0.98
Zondag et al (2024)^29^	Netherlands/Dutch	Maternity care professionals: Community midwives; Hospital-based midwives; Obstetricians	199	Not tested	Natural: 0.76 Medical: 0.74	Not reported
Present study (Lopes et al)	Portugal/European Portuguese	Pregnant women	241	2-factor structure confirmed by EFA and CFA (3 items removed)	Natural: 0.68 Medical: 0.58	Not assessed

Abbreviations: CFA, Confirmatory Factor Analysis; EFA, Exploratory Factor Analysis.

The Portuguese version (BBS-pt) emerges as a promising tool for assessing women’s birth beliefs in both clinical and research contexts. By identifying whether women primarily conceptualize birth as natural or medicalized, the scale can help uncover belief patterns that shape preferences, decision-making, and birth experiences. Such insights can inform more personalized childbirth preparation and maternity care. At the same time, the reduced consistency of the Medical subscale and the exclusion of pain-related items highlight areas for refinement. The weaker Medical dimension may reflect the hybridity of women’s perspectives in Portugal: while medical interventions are widely available and accepted, many women simultaneously endorse birth as a physiological process. This spectrum of beliefs, rather than alignment with a single discourse, may weaken the internal coherence of the Medical subscale.

From a theoretical standpoint, the results suggest that the original two-factor model of the BBS is broadly applicable but not universally stable. Beliefs about childbirth may need to be conceptualized more flexibly, allowing for cultural and experiential variation, particularly regarding pain, women’s sense of autonomy in decision-making, and attitudes toward medical interventions during labor and birth.

Future research should prioritize refinement of the Portuguese version of the BBS. In particular, items related to labor pain may require reformulation to better capture personal beliefs rather than socially normative statements. Cognitive interviews with pregnant women could help clarify how these items are interpreted and guide the development of culturally meaningful wording. Additional psychometric testing with larger and more diverse samples is also needed to confirm the factorial structure and to evaluate measurement invariance across subgroups such as parity, education, and models of maternity care. Furthermore, future studies could explore the development of an additional subdomain addressing beliefs about labor pain, given the ambivalent responses observed in this study. Refining the Medical beliefs subscale by introducing culturally relevant items reflecting contemporary maternity care in Portugal may also improve internal consistency and conceptual clarity. Longitudinal research examining how childbirth beliefs evolve throughout pregnancy and postpartum may further strengthen the scale’s predictive validity and clinical relevance.

Finally, the BBS-pt provides an opportunity for cross-cultural comparisons broadening understanding of how social, cultural, and institutional contexts shape women’s beliefs across healthcare systems. Strengthening this line of research will deepen theoretical knowledge and enhance professionals’ capacity to deliver woman-centered, evidence-based care. For practice, the BBS-pt also offers opportunities to identify ambivalent or conflicting beliefs during antenatal care. Nurse-midwives could use this knowledge to tailor childbirth preparation programs, addressing both the expectation of pain relief and the recognition of pain as meaningful. Acknowledging ambivalence, rather than attempting to “correct” one view at the expense of the other, may foster women’s agency, improve coping, and promote more positive experiences.

### Strengths and Limitations

This study represents the first cross-cultural validation of the Birth Beliefs Scale in a Portuguese-speaking context, conducted with a sample of pregnant women attending childbirth preparation programs. Strengths include the rigorous and systematic adaptation process, the combined use of exploratory and CFA, and the integration of both psychometric and theoretical criteria for item retention.

Several limitations should also be acknowledged. The internal consistency of the Medical subscale was modest, potentially due to the reduced number of retained items and cultural variation in the conceptualization of medicalized childbirth. The removal of items related to labor pain may have narrowed the conceptual scope of the scale and underscores the complexity of assessing pain-related beliefs within a culturally sensitive framework. In addition, the sample was predominantly nulliparous and highly educated, and recruitment from childbirth preparation programs may limit the generalizability of findings to more diverse populations. The absence of test–retest reliability analysis and the cross-sectional design also restrict conclusions about the stability and predictive value of birth beliefs over time. Future studies should address these limitations by recruiting more heterogeneous samples, adopting longitudinal designs, and exploring alternative or expanded item sets, particularly regarding labor pain beliefs.

## Conclusion

Beliefs surrounding childbirth are deeply embedded within cultural, social, and historical frameworks, making childbirth a highly context-sensitive experience. These beliefs shape perceptions of the body, pain, and agency, as well as expectations and behaviors throughout the birth process.

This study adapted and validated the BBS for the Portuguese context, responding to the need for instruments that specifically assess childbirth beliefs, distinct from related constructs such as fear, control, or decision-making. The findings partially support the original two-factor model and provide a culturally grounded basis for assessing how women conceptualize childbirth as a natural or medical event. While some items, particularly those related to labor pain, demonstrated limited psychometric performance and warrant further refinement, the BBS-pt offers a valuable foundation for both research and clinical application. It enables a more precise understanding of childbirth beliefs and their potential influence on women’s preferences, choices, and experiences.

In practice, the BBS-pt can support nurse-midwives and maternity care providers in tailoring education, communication, and support strategies to align with women’s individual values and expectations. Incorporating the assessment of childbirth beliefs into antenatal care may strengthen shared decision-making and contribute to more personalized, empowering, and woman-centered care. This initial version of the BBS-pt thus represents a promising step toward future refinement and longitudinal application.
